# Supplementary material for: Complexome profiling of the Chlamydomonas psb28 mutant reveals TEF5 as an early PSII assembly factor
Source: Plant Cell. 2025 Mar 17;37(6):koaf055. doi: 10.1093/plcell/koaf055 (PMC12164590; doi:10.1093/plcell/koaf055)
Supplement: koaf055_Supplementary_Data [file koaf055_supplementary_data.zip › psb28_interactive.zip!/plots/intensity/qualityPlots/peptides/TIC110_Cre10.g452450.t1.2.html]

### Cre10.g452450.t1.2

Trivial name: TIC110  
SeqCoverage [median]: 51.40  
Mass: 115.13 kDa  
  
 

| Sequence | MissedCleavage | Mass [Da] | wtIntensity | muIntensity | Score |
| --- | --- | --- | --- | --- | --- |
| AAPVDPGVK | 0 | 852.47 | 1231.46 | 838.71 | 69.20 |
| AAPVDPGVKEVASYGVAAVAAAATAYGAVQAK | 1 | 2972.56 | 669.84 | 0.00 | 42.66 |
| AFPYPLPK | 0 | 931.52 | 5063.21 | 6559.81 | 81.38 |
| AQASEVMR | 0 | 890.43 | 9880.50 | 14335.17 | 169.09 |
| DILGLADEDAAPVHIEVGRR | 1 | 2145.12 | 890.15 | 9135.93 | 59.43 |
| EFASAIK | 0 | 764.41 | 0.00 | 617.87 | 71.72 |
| EFASAIKK | 1 | 892.50 | 0.00 | 1440.71 | 67.17 |
| EGFETK | 0 | 709.33 | 616.35 | 1525.90 | 89.90 |
| EISLKDDLEPAMR | 1 | 1515.76 | 682.35 | 6431.89 | 138.02 |
| ELAEMLMK | 0 | 963.48 | 11669.69 | 16449.28 | 98.42 |
| ELLPAALQLPENK | 0 | 1434.81 | 5493.54 | 8870.12 | 124.10 |
| ETGDKVLK | 1 | 888.49 | 381.91 | 595.10 | 93.99 |
| ETQGLRDEVSAK | 1 | 1331.67 | 13801.23 | 24262.33 | 92.11 |
| FRGYVSQAQK | 1 | 1182.61 | 7295.07 | 11168.60 | 125.75 |
| FRPEAALELHR | 1 | 1337.72 | 1708.53 | 17617.18 | 95.40 |
| GEYTEEER | 0 | 1011.41 | 1997.50 | 2837.11 | 89.14 |
| GEYTEEERK | 1 | 1139.51 | 0.00 | 616.19 | 75.38 |
| GGALPADR | 0 | 755.39 | 2910.91 | 6874.19 | 92.47 |
| GGALPADRHFLR | 1 | 1308.71 | 0.00 | 3434.22 | 78.69 |
| GGLTDADAEDLKR | 1 | 1359.66 | 25062.66 | 39419.90 | 105.03 |
| GRDMKDVFR | 2 | 1122.56 | 0.00 | 1343.27 | 68.89 |
| GRGGLTDADAEDLKR | 2 | 1572.79 | 561.21 | 5774.43 | 93.48 |
| GYVSQAQK | 0 | 879.45 | 1745.82 | 5455.05 | 161.68 |
| IIVKEEVSTR | 1 | 1172.68 | 32184.46 | 78713.66 | 133.91 |
| IQYLDEMR | 0 | 1066.51 | 0.00 | 3174.77 | 100.45 |
| IQYLDEMRTQLGLTK | 1 | 1807.95 | 0.00 | 5363.47 | 56.48 |
| IYGQFLESVIPTGDTQLKGDEAIK | 1 | 2621.36 | 1260.58 | 0.00 | 63.52 |
| KSIQATR | 1 | 802.47 | 0.00 | 866.03 | 89.05 |
| KVTDGSGETDVSYFK | 1 | 1631.77 | 307.17 | 2118.09 | 60.92 |
| LDSEVAVDVMAR | 0 | 1303.64 | 0.00 | 4960.34 | 150.10 |
| LEVYGSSSAAEDGK | 0 | 1411.65 | 1593.14 | 4327.46 | 128.36 |
| LFDETAAEVVK | 0 | 1220.63 | 12684.79 | 43458.87 | 142.83 |
| LFDETAAEVVKEAAR | 1 | 1647.85 | 1329.92 | 18984.63 | 75.67 |
| LIYVSQVVFGDQK | 0 | 1494.81 | 2919.59 | 16371.14 | 184.27 |
| LLKEEVTSGR | 1 | 1130.63 | 23872.09 | 36209.87 | 110.52 |
| LLQFNALMVTPLLDK | 0 | 1714.97 | 1242.52 | 0.00 | 119.74 |
| LLQFNALMVTPLLDKVK | 1 | 1942.13 | 498.15 | 0.00 | 52.82 |
| LNSLADVLGMSQQEVMSAQSDLAEQAYK | 0 | 3025.44 | 677.80 | 0.00 | 42.47 |
| MASLLESR | 0 | 905.46 | 6077.01 | 19108.87 | 183.90 |
| MASLLESRR | 1 | 1061.57 | 0.00 | 1109.25 | 68.02 |
| MLLVQAVSQFR | 0 | 1290.71 | 6685.34 | 29879.46 | 170.47 |
| NAVFEK | 0 | 706.36 | 0.00 | 1044.25 | 76.13 |
| QFLEAR | 0 | 762.40 | 1295.21 | 2998.50 | 84.06 |
| RGRGGLTDADAEDLKR | 3 | 1728.89 | 0.00 | 760.21 | 80.63 |
| RSGGLADDDEDFF | 1 | 1442.60 | 911.04 | 5215.16 | 110.84 |
| RTFNLNDAQIFVAR | 1 | 1663.88 | 0.00 | 3633.04 | 51.64 |
| SGGLADDDEDFF | 0 | 1286.49 | 783.56 | 0.00 | 84.57 |
| SGPLNEEK | 0 | 872.42 | 2469.45 | 4490.55 | 128.00 |
| SGPLNEEKIQYLDEMR | 1 | 1920.93 | 2126.72 | 10549.99 | 144.73 |
| SIQATRGEYTEEER | 1 | 1667.78 | 1979.60 | 3619.40 | 102.28 |
| TFNLNDAQIFVAR | 0 | 1507.78 | 2302.27 | 6253.77 | 184.36 |
| TFNLNDAQIFVARR | 1 | 1663.88 | 0.00 | 1047.50 | 35.74 |
| TGGSVDQLLEEVTRR | 1 | 1658.86 | 2346.15 | 17856.72 | 98.69 |
| TQLGLTK | 0 | 759.45 | 2951.99 | 9916.90 | 119.17 |
| TVEAHLSK | 0 | 883.48 | 1770.29 | 1956.45 | 114.50 |
| VLELHK | 0 | 737.44 | 1123.12 | 5767.07 | 126.04 |
| VQLETAGK | 0 | 844.47 | 0.00 | 347.49 | 58.98 |
| VTDGSGETDVSYFK | 0 | 1503.67 | 5482.99 | 7005.25 | 208.33 |
| YGINFQKDELDGLKR | 2 | 1794.93 | 3587.61 | 57062.78 | 120.30 |
| YGSEDDLVSGLGLVTIHGGALDVEGK | 0 | 2600.30 | 1879.54 | 0.00 | 150.06 |
|  |  |  | 214004.0 | 589773.9 |  |
